# TRPM2 channels in mitochondrial dynamics and cancer

**DOI:** 10.18632/oncotarget.21391

**Published:** 2017-09-30

**Authors:** Asipu Sivaprasadarao, Nada Abuarab, Fangfang Li

**Affiliations:** Asipu Sivaprasadarao: School of Biomedical Sciences, Faculty of Biological Sciences, Multidisciplinary Cardiovascular Research Centre, University of Leeds, Leeds, UK

**Keywords:** mitochondrial dynamics, TRPM2, reactive oxygen species, calcium, zinc

In most cell types, mitochondria exist as a branched, tubular network. The structural and functional integrity of the network is maintained by its continuous fission and fusion [1, 2]. Fission segregates functional (polarised) parts of the network from dysfunctional (depolarised) parts, enabling the clearance of dysfunctional mitochondria by mitophagy. Fission is catalysed by Drp1 (dynamin-related protein1), which is recruited to mitochondria from the cytoplasm. Fusion joins the functional mitochondria with each other and with the existing network. Fusion is mediated by Mitofusin-1/2 (MFN-1/2) and OPA1 (Optic atrophy type 1) which catalyse the merger of the outer and inner membranes of mitochondria, respectively. These processes, collectively known as ‘mitochondrial dynamics’, are essential to sustain the bioenergetic, biosynthetic and signalling functions of mitochondria. Failure to balance the fission and fusion activities is implicated in a number of age-related and other oxidative stress-associated human diseases, including cancer [1, 2].

Mitochondrial dynamics not only contribute to mitochondrial quality control, but play a crucial role in normal cell division [1]. In the G1/S phase of the cell cycle, mitochondria exist as a branched network to ensue efficient ATP supply for cell proliferation. In the S/G2 phase, Drp1 is phosphorylated by cyclin-dependent kinase-1, promoting its recruitment to the mitochondrial network, and the subsequent fragmentation. Thus by reducing the complexity of the mitochondrial network, fission enables equal distribution of mitochondria between the daughter cells. It is therefore not surprising that genetic inactivation of any of the proteins associated with mitochondrial dynamics is embryonic lethal [1].

Mitochondrial dynamics have been implicated in cancer cell proliferation as well as migration. In most cancer cells, mitochondria are fragmented [1, 2]. This was attributed to the increased expression or activation of Drp1, coupled with the downregulation of MFN-2. Inhibition of Drp1 and overexpression of MFN2 prevented abnormal cell proliferation, and intriguingly also increased spontaneous apoptosis [1, 2]. A recent study demonstrated that stimulation of oncogenic K-Ras induces Erk1/2-mediated phosphorylation of Drp1 and subsequent mitochondrial fragmentation in pancreatic cancer [1].

Mitochondrial dynamics play a role in tumour cell migration [1]. Increased Drp1 activity enables smaller mitochondria to move along the microtubules to the leading edge (lamellipodia) of migrating cells. Presence of adequate number of mitochondria at the leading edge ensures ATP supply to processes (focal adhesion and actin dynamics) essential for cell migration. Inhibition of Drp1 prevented mitochondria migration as well as cell migration, suggesting that fission is a prerequisite for efficient translocation of mitochondria [1].

Thus Drp1 plays a central role in mitochondrial fragmentation linked to most important hallmarks of cancer: abnormal cell proliferation and unwarranted cell migration [1]. Increased mitochondrial fragmentation is also a fundamental feature of a number of other human diseases including diabetes, and cardiovascular (e.g. ischemia) and neuronal (Parkinson’s, Alzheimer’s and stroke) diseases [2]. Thus there has been considerable focus on Drp1. Drp1 activity and its recruitment to mitochondria are regulated by a remarkable number of signals that modify the protein in multiple ways (phosphorylation, ubiquitination, nitrosylation, acetylation and sumoylation) [1]. Recent studies have added three more players to the already known long list of regulators of mitochondrial fission: the TRPM2 (transient receptor potential melastatin2) ion channel, lysosomes and Zn^2+^ [3, 4].

The authors of these new studies showed that inhibition of TRPM2 channels prevents oxidative stress (high glucose or free fatty acid)-induced mitochondrial fragmentation in human endothelial cells and pancreatic β-cells, revealing a previously unappreciated mechanism [3, 4]. Oxidative stress increases the cellular levels of reactive oxygen species (ROS), leading to the activation of TRPM2 channels, extracellular Ca^2+^ entry, Ca^2+^-induced lysosomal Zn^2+^ release and the subsequent entry of free Zn^2+^ into mitochondria. Rise in mitochondrial Zn^2+^ causes a loss of mitochondrial membrane potential, resulting in mitochondrial Drp1 recruitment and fragmentation. Given that ROS increase is a hallmark of most cancer cells [5], and ROS-sensitive TRPM2 channels are upregulated in some cancers (http://www.proteinatlas.org/ENSG00000142185-TRPM2/cancer), it seems reasonable to speculate that a similar mechanism might contribute to the increased mitochondrial fragmentation in cancer cells. If true, this mechanism could provide the fundamental missing link between increased ROS and mitochondrial fragmentation in cancer cells.

Several recent studies provide circumstantial support for such speculation. RNAi-mediated silencing of TRPM2 channels prevents prostate cancer cell (PC-3) proliferation [6] and migration [5]. Ca^2+^ is well known for its signalling role in cancer cell proliferation and migration [7]. Zn^2+^ has also been implicated in oxidative stress-induced cancer cell migration [5].

Mitochondrial dynamics is an attractive drug target for cancer as well as a host of other diseases, mentioned above. Many of the players in mitochondrial dynamics are deemed unsuitable or less safe for development of therapeutics. However, despite the fact that genetic inactivation of Drp1 is embryonic lethal [1], Drp1 has been targeted for drug development. Indeed pharmacological inhibition of Drp1 with mdivi1 showed considerable promise in preclinical studies of cancer and other diseases [8]. Remarkably, however, the beneficial effect of mdivi1 does not appear to be conferred by its ability to inhibit Drp1, but rather owing to the inhibition of mitochondrial complex 1 [8]. Given ion channels are excellent drug targets, the new findings imply that targeting TRPM2 channels for cancer and other mitochondrial dynamics-associated diseases may represent a promising therapeutic approach.

## References

[1] Senft D (2016). Curr Opin Cell Biol.

[2] Archer SL (2013). N Engl J Med.

[3] Abuarab N (2017). Sci Signal.

[4] Li F (2017). Cell Death Differ.

[5] Li F (2016). J Cell Sci.

[6] Zeng X (2010). Prostate Cancer Prostatic Dis.

[7] Prevarskaya N (2011). Nat Rev Cancer.

[8] Bordt EA (2017). Developmental Cell.

